# Polarimeters for the Detection of Anisotropy from Reflectance

**DOI:** 10.3390/mi15060794

**Published:** 2024-06-17

**Authors:** Shuji Kamegaki, Zahra Khajehsaeidimahabadi, Meguya Ryu, Nguyen Hoai An Le, Soon Hock Ng, Ričardas Buividas, Gediminas Seniutinas, Vijayakumar Anand, Saulius Juodkazis, Junko Morikawa

**Affiliations:** 1 School of Materials and Chemical Technology, Tokyo Institute of Technology, Ookayama, Meguro-ku, Tokyo 152-8550, Japan; kamegaki.s.aa@m.titech.ac.jp; 2 Optical Sciences Centre, ARC Training Centre in Surface Engineering for Advanced Materials (SEAM), Swinburne University of Technology, Hawthorn, VIC 3122, Australia; zkhajehsaeidimahabad@swin.edu.au (Z.K.); ale@swin.edu.au (N.H.A.L.); soonhockng@swin.edu.au (S.H.N.); rbuividas@swin.edu.au (R.B.); gseniutinas@swin.edu.au (G.S.); 3 Aerostructures Innovation Research Hub (AIR Hub), Swinburne University of Technology, John St, Hawthorn, VIC 3122, Australia; 4 National Metrology Institute of Japan (NMIJ), National Institute of Advanced Industrial Science and Technology (AIST), Tsukuba Central 3, 1-1-1 Umezono, Tsukuba 305-8563, Ibaraki, Japan; ryu.meguya@aist.go.jp; 5 Institute of Physics, University of Tartu, W. Ostwaldi 1, 50411 Tartu, Estonia; vijayakumar.anand@ut.ee; 6 WRH Program International Research Frontiers Initiative (IRFI), Tokyo Institute of Technology, Nagatsuta-cho, Midori-ku, Yokohama 226-8503, Kanagawa, Japan; 7 Laser Research Center, Physics Faculty, Vilnius University, Saulėtekio Ave. 10, 10223 Vilnius, Lithuania; 8 Research Center for Autonomous Systems Materialogy (ASMat), Institute of Innovative Research, Tokyo Institute of Technology, 4259 Nagatsuta-cho, Midori-ku, Yokohama 226-8501, Kanagawa, Japan

**Keywords:** polarimetry, anisotropy, birefringence, four-polarisation method

## Abstract

Polarimetry is used to determine the Stokes parameters of a laser beam. Once all four $S_{0,1,2,3}$ parameters are determined, the state of polarisation is established. Upon reflection of a laser beam with the defined *S* polarisation state, the directly measured *S* parameters can be used to determine the optical properties of the surface, which modify the *S*-state upon reflection. Here, we use polarimetry for the determination of surface anisotropies related to the birefringence and dichroism of different materials, which have a common feature of linear patterns with different alignments and scales. It is shown that polarimetry in the back-reflected light is complementary to ellipsometry and four-polarisation camera imaging; experiments were carried out using a microscope.

## 1. Introduction

Stokes polarimetry [1] is a powerful optical characterisation technique for the determination of the state of light as incoherent or coherent [2]. Linearly and circularly polarised illumination of materials can be used for the identification of chirally active biological and organic materials [3]. Optical activity and dextrorotatory (+ or D)- and levorotatory (− or L)-specific rotation affect the state of polarisation of transmitted light, which is measured by a polarimeter. Once $S_{1,2,3}$ is known, it sets a coordinate basis for a convenient presentation of the light state on the Poincaré sphere: linear polarisations occur on the equator and circular polarisations occur on the poles, with an arbitrary state at other locations on the surface (this can be considered as a mixture of circular and linear polarisation). When the incident polarisation is known, i.e., the Stokes vector is defined, transmitted or reflected/scattered light can be analysed via polarimetry. If illumination is from natural light, it can also be represented by its polarisation state using the Stokes vector [4]. This hypothesis is proven by the fact that Viking seafarers navigated the northern Atlantic using polarisation of scattered sunlight in low-visibility environments due to fog and clouds [5]. The presence of strong magnetic fields and scattering can be inferred from astronomic polarimetry [6]. Changes in the Stokes vector represent light–matter interactions in terms of a change in polarisation due to dichroism, birefringence, or/and scattering [7]. Also, strong transverse (along the *r* radial coordinate) gradients of the refractive index *n* or permittivity $\varepsilon =n^{2}$ cause transverse alterations in polarisation as $\frac{𝜕E}{𝜕r}\propto -E\frac{𝜕\ln\varepsilon }{𝜕r}$ along propagation (along the *z*-axis) [8]. This can be utilised in remote sensing using optical detection, nanofabrication by ablation and structural modifications [9], or light localisation and polymerisation inside nano-gaps [10]. A recent trend is to miniaturise Stokes polarimeters using waveplates, gratings, and metamaterials [11] or 2D materials [12]. For the transmission geometry, very simple polarimeters with high-precision detection of polarisation changes have been proposed [13,14]. Imaging polarimeters based on splitting the beam into three bases of two linear and circular polarisations without polarisation projection allows for the use of all available light intensities for Stokes polarimetry [15].

The reflection geometry has the largest practical value due to the simplicity of the experiment and its remote character, e.g., from a satellite in orbit or a drone/plane. Even though the exact properties of materials are not known due to complex compositions and structures, the anisotropy of patterns can be recognised. Importantly, using polarisation analysis, these anisotropies can be recognised at a resolution up to twenty times below the diffraction limit of the used focusing optics, as was demonstrated for IR transmittance [16]. It was shown that reflection from gold half-mirrors at a $45^{\circ }$ degree of incidence, which is typical in optical microscopy, has to be measured via polarimetry for a reliable description of the polarisation state of the reflected light rather than relying on analytical solutions based on real and imaginary parts of the refractive index n+ik, which affect the phase of the reflected light [17]. Reflection from a gold half-mirror preserves the degree of polarisation of the incident light [17]; however, the phase of the reflected light is dependent on the complex refractive index n+ik at the wavelength of incidence.

Here, we determine the optical anisotropy related to the phase delay δ in back-reflection geometry using laser polarimetry. The principle is shown using a microscope; however, its generic principle implies that measurements in industrial and remote sensing settings with illumination detection are possible. Different patterns of dielectric and conductive materials with linearly aligned structures at a range of different feature sizes and complexities were used to test polarimetry in reflection. Four-polarisation (4-pol.) camera images at the same four angles are compared with four-angle polarimetry measurements from a single point of illumination. This study is aimed at testing the capability of detecting the structural anisotropy of the reflecting surface via polarimetry, which is a simpler method compared with 4-pol. imaging and ellipsometry. Polarimetry can be implemented in field conditions.

## 2. Methods and Materials

### 2.1. Polarimetry Method

**Figure 1:**
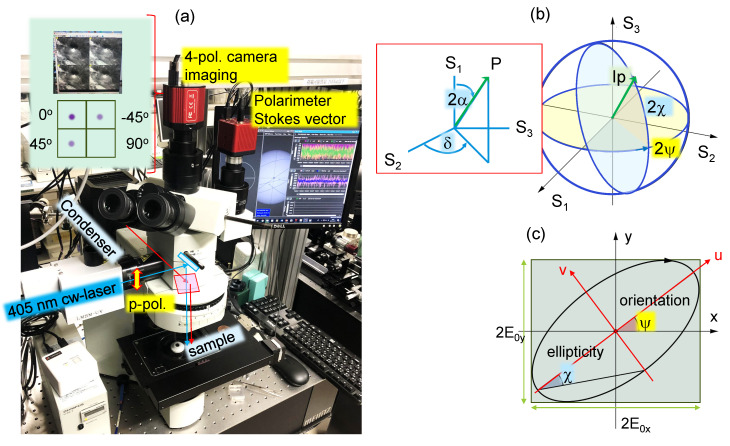
(**a**) Setup used in this study of reflection polarimetry using a four-polarisation camera as an imaging device together with a polarimeter. Poincaré sphere presentation (**b**) of the Stokes vector $\left(S_{1},S_{2},S_{3}\right)$ and polarisation ellipse (**c**). The total incident intensity is *I* and the degree of polarisation *p* is 0≤p≤1; we used linearly polarised p=1 (p-pol. or vertical) laser light. The inset (**b**) shows the equivalent observable polarisation sphere (OPS) presentation.

Three Stokes parameters $S_{1,2,3}$ and the degree of polarisation *p* were directly measured using a polarimeter (PAX1000, Thorlabs, Newton, MA, USA), which works on the principle of rotating a λ/4 waveplate and a fixed linear polariser in front of the detector; $S_{0}=1$ is the incident intensity. A convenient visualisation of the Stokes vector is a Poincaré sphere, which is linked to the in-plane presentation of a polarisation ellipse (Figure 1). The orientation (azimuth) angle ψ (0≤ψ≤π) and the ellipticity angle χ (−π/4<χ≤π/4) are calculated (for the normalised case $S_{0}=1$), respectively, by [2]:

(1) $$\tan\left(2\psi \right)=\frac{S_{2}}{S_{1}},$$

(2) $$\tan\left(2\chi \right)=\frac{S_{3}}{\sqrt{S_{1}^{2}+S_{2}^{2}}}.$$

By the introduction of an auxiliary angle α (0≤α≤π/2) defined in the principle (x,y)-plane (s,p-polarisation) $\tan\alpha =E_{0y}/E_{0x}$, a set of equations is used to determine α and phase retardance $\delta =\delta_{y}-\delta_{x}$ from the determined ψ and χ [2]:

(3) tan(2ψ)=tan2α×cosδ,

(4) sin(2χ)=sin2α×sinδ.

The pair of (α,δ) can be solved from the measured Stokes vector and hence from (ψ,χ).

Analytical expressions are also available as follows from an equivalent to the Poincaré sphere presentation, namely, the observable polarisation sphere (OPS), upon conversion of coordinates $\left(S_{1},S_{2},S_{3}\right)$ into (cos(2α),sin(2α)cosδ,sin(2α)sinδ); see the OPS inset in Figure 1. Since $S_{2}=2E_{x}E_{y}\cos\delta /S_{0}$ and $S_{3}=2E_{x}E_{y}\sin\delta /S_{0}$, we obtain $\tan\delta =S_{3}/S_{2}$ (0≤δ≤2π). This provides direct analytical access to δ from the measured Stokes vector. With a few steps, Equations (3) and (4) yield $\cos\left(2\alpha \right)=S_{1}$ (0≤2α≤π). These analytical expressions were used for the determination of α and δ. We used a linearly polarised laser beam, which is useful for fast assessment of the Stokes parameters in the reflected beam. Namely, from $S_{2}$ and $S_{3}$ expressions, once $S_{1}=1$ (horizontal x-pol.), $S_{2}=S_{3}=0$ is expected since the product of $E_{y}=0$ and $E_{x}=1$ defines $S_{2,3}$. This rule was observed in experiments.

### 2.2. Imaging

Samples were imaged and the polarisation of reflected light was analysed using a 4-pol. camera (CS505MUP1, Thorlabs). For the linear polarisation of incidence, it can only be used for the determination of $S_{1}$ and $S_{2}$ (this study). With the prepared circular-polarised incidence, all components of the Stokes vector are accessible [18]. The 4-pol. camera and polarimeter were set up on an Olympus BX51 microscope (Olympus, Ishikawa, Japan) for simultaneous operation (Figure 1); a Xe lamp condenser was used for top illumination of the sample with white light. Importantly, the circular broadband polarisers used in front of the imaging cameras in modern microscopes have to be removed to use the 4-pol. camera, as they were in this study (one quadrant of a 4-pol. camera image should be dark upon linearly polarised illumination). This was achieved for the alignment of the 4-pol. camera by setting equal intensities of ±π/4 quadrants on the histogram. A side port was used to introduce a 405 nm laser beam in the reflection geometry.

For structural surface characterisation, field-emission scanning electron microscopy (SEM) was used (Raith150TWO, Raith GmbH, Dortmund, Germany).

### 2.3. Samples

Samples with linear patterns were selected for study: a carbon-fiber-reinforced polymer (CFRP) and a polymerised micro-grating. Samples were commercial plates of polyetheretherketone (PEEK) composites strengthened with carbon fibers weighing 66% (denoted as CF66 PEEK), featuring a unidirectional alignment of fibers with a thickness of 184μm. The reason behind the extensive use of carbon fibers as a reinforcing material in PEEK-based composites is the robust interaction at the interface between the carbon fibers and the PEEK matrix [19]. Variable-angle spectrometric ellipsometry (VASE) in the visible to near-IR spectral range was carried out on CFRP samples using J.A. Woollam’s ellipsometer.

The laser-polymerised rods, which are a few micrometers in width and height, form a grating on the cover glass substrate. The polymer sample was Au-coated. Samples with complex, azimuthally oriented, sub-wavelength-scale patterns were constructed on Si via fs laser ablation and nanofabricated on a membrane of polycrystalline diamond.

## 3. Results and Discussion

Polarimetry as a single-point measurement at normal incidence was used in this study. The experimentally measured Stokes vector was then used for the determination of α; hence, the ratio of $E_{0y}/E_{0x}$, which is equivalent to $E_{p}/E_{s}$ in s,p-polarisation, is defined in the plane of incidence. Notably, the reflectance of s- and p-pol. at normal incidence is the same ($r_{s}\equiv r_{p}$) for the E-field components from a uniform material. However, anisotropy of light scattering can be present from uneven surfaces for the two perpendicular orientations of the incident E-field. This is explored in the current study. If only anisotropy in the scattering or absorbance within the skin depth $l_{s}\equiv 1/\alpha_{abs}={\left[4\pi k/\lambda \right]}^{-1}$ is present, the polarisation should be in the principle axis plane (x,y), i.e., (s,p). Here, $\alpha_{abs}$ is the absorption coefficient [${\mathrm{cm}}^{-1}$] and *k* is the imaginary part of the complex refractive index $\tilde{n}=n+ik$.

Similarly, δ or phase retardance due to birefringence within the skin depth or normal/anomalous dispersion regions around the absorption line (at a fixed wavelength), as well as depolarisation due to scattering, can appear as retardance due to birefringence Δn: $\delta =\frac{2\pi \Delta n}{\lambda }$. As for usual transmittance measurements, birefringence causes a projection of light onto two orthogonal optical axes resulting in a larger ellipticity angle χ and its orientation ψ; for δ=π/2, a circular polarisation is obtained and the ellipse becomes a circle (Figure 1). Left or right circular polarisation depends on the sign of χ, with positive being LHC or anti-clockwise (looking into the beam) and negative being RHC or clockwise.

When a short wavelength is used, the scattering is strong $\propto \lambda^{4}$ and the skin depth is shallow $l_{s}\approx \lambda /4$. We used a λ=405 nm cw laser to determine Stokes vectors from normal reflection from different materials of industrial relevance: a carbon-fiber-reinforced polymer (CFRP) and laser-polymerised grating patterns. Experiments were carried out at four sample orientation angles using a polarimeter. The same four angles are used to reveal the orientation and anisotropy using linear polarisers in a 4-pol. camera (Figure 1a).

### 3.1. Linear Patterns at Different Degrees of Alignment

**Figure 2:**
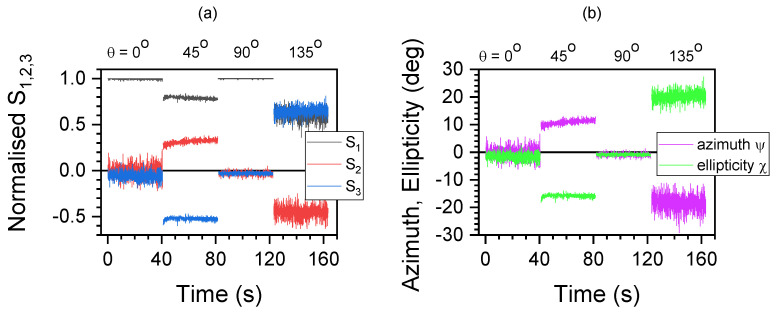
Polarimetry of a CFRP at 405 nm. (**a**) Normalised Stokes vector $\left(S_{1},S_{2},S_{3}\right)$ at different sample orientation angles θ measured for 40 s. (**b**) Azimuth ψ and ellipticity χ of the polarisation ellipse over time. Laser power: 55 mW.

Figure 2a shows the Stokes parameters $S_{1,2,3}$ of the laser beam with a 405 nm wavelength reflected from a CFRP at different incident linear polarisations, e.g., $\theta =0^{\circ }$ or the with horizontal x-direction coinciding with the orientation of fibers in the CFRP. The corresponding azimuth and ellipticity angles are calculated as shown in (b). When the reflected linearly polarised light $S_{1}$ is orientated along the fibers $\theta =0^{\circ }$, the signal is strong (∼1). In this case, there are no ±π/4-tilted or circularly polarised components, which makes $S_{2}\approx S_{3}\approx 0$. The reflected intensity is lower due to the stronger absorption/scattering as compared with vertical polarisation $\theta =90^{\circ }$. When the incident polarisation is θ=±π/4, there are $S_{2}$ and $S_{3}$ components. This is consistent with reflection from a form-birefringent surface structure, which induces phase differences between two perpendicular polarisation components. This also makes it appealing to present the azimuth and ellipticity angles ψ,χ.

**Figure 3:**
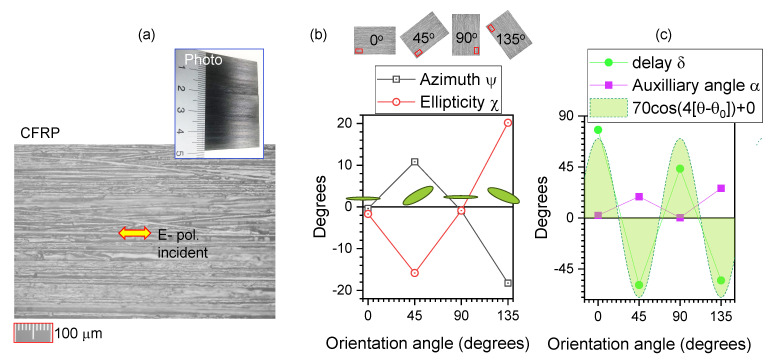
(**a**) Microscopy image in reflection off a carbon-fiber-reinforced polymer (CFRP) with apparent prevalent horizontal alignment of fibers. (**b**) The averaged azimuth and ellipticity angles ψ,χ, respectively, calculated from the measured Stokes vector at different orientation angles θ of the sample; $\theta =0^{\circ }$ corresponds to the horizontal *x*-axis (see raw data in Figure 2). (**c**) The delay δ and auxiliary angle α vs. θ; the fit by $Amp\times \cos(4\left[\theta -\theta_{0}\right]+\mathrm{Offset})$ is plotted, where $\theta_{0}=0$, Offset=0, and $Amp=70^{\circ }$. Illumination with a cw laser at 405 nm.

**Figure 4:**
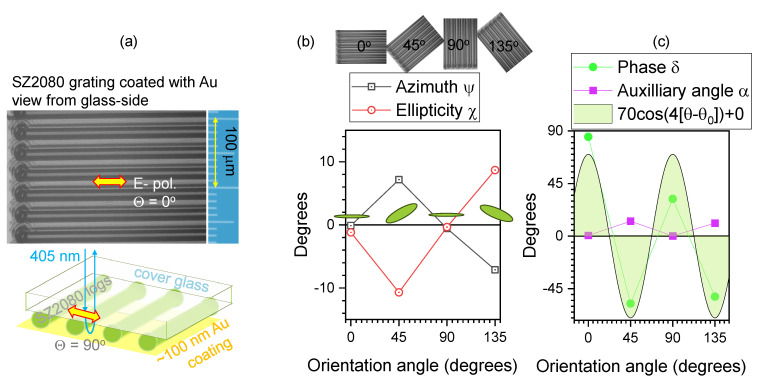
(**a**) Microscopy image in the reflection off a SZ208$0^{\mathrm{TM}}$ resist grating polymerised by fs laser direct writing. The schematic drawing shows the sample’s structure: polymerised rods protruding ∼5–7 μm out of the cover glass (∼150 μm thickness) and coated with ∼100 nm of Au. (**b**) The azimuth and ellipticity angles, ψ,χ, respectively, calculated from the measured Stokes vector at different orientation angles θ; $\theta =0^{\circ }$ corresponds to the horizontal *x*-axis. (**c**) The phase retardance δ and auxiliary angle α vs. θ; the fit by $Amp\times \cos(4\left[\theta -\theta_{0}\right]+\mathrm{Offset})$ is plotted, where $\theta_{0}=0$, Offset=0, and $Amp=70^{\circ }$. Illumination with a cw laser at 405 nm.

Figure 3 and Figure 4 show α and δ calculated from the Stokes vector for reflection. The common feature is that the linear structures are 7–15 μm in diameter, while the focal spot is considerably smaller (1.22λ/NA≈3.8μm for NA=0.13 focusing). The CFRP sample has a black appearance, with the carbon fibers aligned preferentially in one direction. The polymerised SZ208$0^{\mathrm{TM}}$ resist grating, with a period of Λ≈30μm, was coated by a ∼100 nm Au film via sputtering. When measured from the Au side, the reflected polarisation was the same as that of the incident beam regardless of the orientation; the same was observed for a flat Au mirror or a Si wafer surface. Figure 4 shows the results when the laser was irradiating the sample from the uncoated glass side. In both cases, the reflected Stokes parameters had a qualitatively similar behaviour. When the delay δ was high and approaching π/2, which would correspond to the birefringence defining a λ/4-waveplate condition, the auxiliary angle α→0, which corresponds to a negligible $E_{y}\to 0$ component. Thus, no circular polarisation was formed in reflection; i.e., the same linear polarisation as that of incidence ($E_{x}$) was observed and only the phase was delayed upon reflectance. When light back-reflects from an interface with a higher-refractive-index material (impinging from air n=1), a phase change of π occurs. In both cases of linearly aligned patterns (Figure 3c and Figure 4c), the qualitative 4-pol. fit was the same for the orientation $\theta_{0}$, amplitude and offset; since there are only three fit parameters, four points and θ=0,±π/4,π/2 are enough. Interestingly, upon reflection from optically flat absorbing materials, such as 100 μm thick kapton or ∼1 cm epoxy puck, there was a recognisable ellipticity angle χ. This can be attributed to light scattering and depolarisation in subsurface regions.

**Figure 5:**
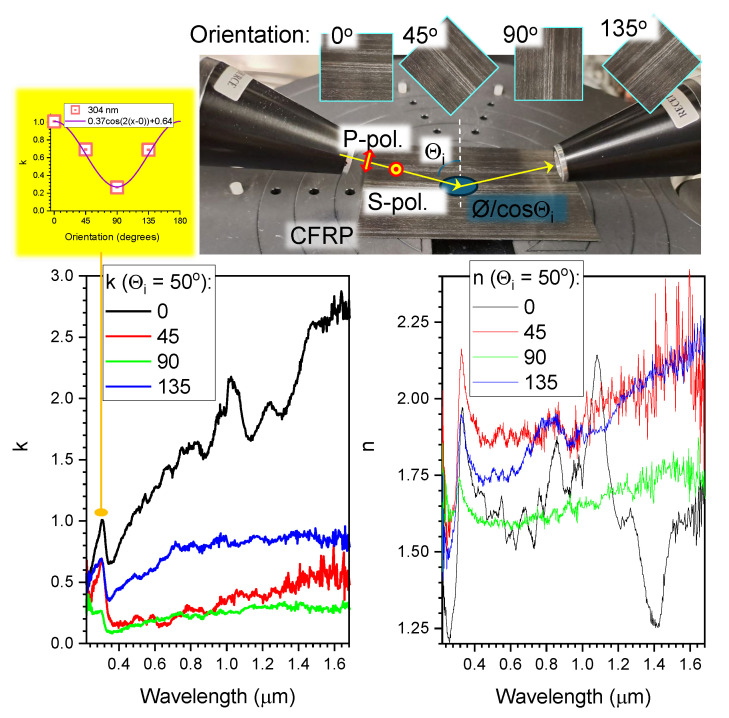
Ellipsometry-based determination of the refractive index (n(λ)+ik(λ)) from spectra in the visible-to-IR wavelength range; the angle of incidence was $\theta_{i}=50^{\circ }$. The top photo shows the geometry of measurements. The absorption peak at 304 nm in k(λ) was selected for fitting by $Amp\times \cos(2\left(\theta -\theta_{0}\right))+\mathrm{Offset}$. This fit implies a dichroism-based anisotropy.

Figure 5 shows the ellipsometry spectra for complex refractive indexes n(λ)+ik(λ). At the selected absorbance peak of 304 nm, the angular dependence of k(θ) was fitted by the Amp×cos(2(θ−0))+Offset function. It has a twice lower angular frequency 2θ (typical for absorbance with folding at π in transmission) compared with the polarimetry fit at 405 nm, which had 4θ dependence (typical for birefringence in transmission). The phase difference/delay δ may result from reflection from a surface which has anisotropic features and a complex composition (e.g., carbon fibers and a polymer composite matrix). Upon reflection, there is a π phase shift when light travels from a low- to a high-index *n* material and is reflected (typical for incidence from air). For absorbing surfaces, this may be different when the real part of permittivity (epsilon) $\varepsilon \equiv {\left(n+ik\right)}^{2}=\left(n^{2}-k^{2}\right)+i2nk$ is less than 1 (like that of air). In Figure 5, such a spectral region is the λ>1.2μm region, where $0<(n^{2}-k^{2})<1$. No phase change upon reflection occurs in this region, called epsilon-near-zero (ENZ), especially for the sample orientation when the polarisation of incident light is aligned to the carbon fibers at $\theta \approx 0^{\circ }$.

In ellipsometry data of the CFRP in the visible–IR range, the 2θ-anisoptropy typical for absorbance is dominant. For 405 nm illumination in reflection at the normal incidence, the CFRP has a low *k* and a large *n*; hence, Re(ε)>1 and this will define the phase change upon reflection (Figure 3). This phase change has a 4θ-dependence typical for the phase delay δ. Light scattering is dominant for the polarimetry measurement, since scattering is related to *n* and hence to δ, unlike absorbance and its dichroism, which are related to *k*.

The reflected laser beam from electrically conductive mirror surfaces maintained its linear polarisation as expected [17]. For metals described with the Drude model, where permittivity $\varepsilon \equiv {\left[n\left(1-i\kappa \right)\right]}^{2}$ [2], κ is used to make a distinction from the above used *k* when the definition of the refractive index is (n+ik). According to the Drude model, $\varepsilon =\varepsilon^{\prime }-i\left[4\pi \sigma /\omega \right]$, where σ is the conductivity and ω=2πν is the cyclic frequency. Hence, from the real and imaginary parts of the permittivity, $\varepsilon^{\prime }=n^{2}\left(1-\kappa^{2}\right)$ and $\varepsilon^{\prime \prime }=n^{2}\kappa =\sigma /\nu$. Depending on the wavelength λ=c/ν, the actual phase change upon reflection from the surface is defined by the real part of the effective permittivity $\varepsilon^{\prime }=n^{2}\left[1-{\left(\frac{\sigma }{n^{2}\nu }\right)}^{2}\right]$.

### 3.2. Azimuthaly Changing Orientation: Laser Ablated Ripples

**Figure 6:**
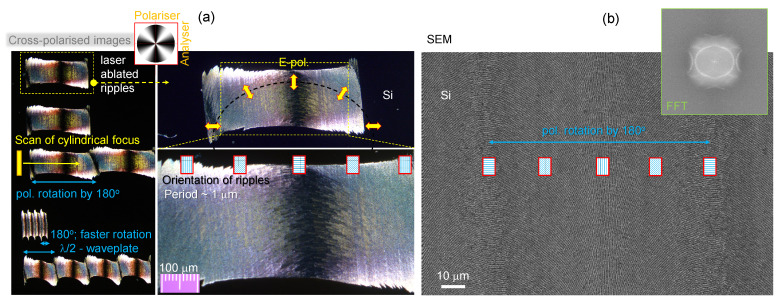
(**a**) Cross-polarised reflection images of ripples induced by fs laser (1030 nm/230 fs) ablation on a Si wafer at different magnifications; the Maltese cross cross-polarised image is shown in the top inset. The laser beam was formed using a cylindrical lens, and polarisation was rotated along the scan direction using a λ/2-waveplate. (**b**) SEM image of azimuthally changing ripples: 1030 nm, 100 kHz, 10 pulses per micrometer, scan v=0.1 mm/s. Focusing: 5 mm diameter beam, f=+80 mm cylinder lens at 180 mm from the objective lens with numerical aperture NA=0.26. Polarisation rotation at a speed of $2^{\circ }$ per micrometer of the linear scan. The fast Fourier transform (FFT) image is shown in the inset, with the main intensity at Λ≈0.80±0.06μm and the period along the scanning direction slightly smaller than the one perpendicular to it.

The linear-grating-like patterns investigated by polariscopy in Section 3.1 were larger than the focal diameter. Here, we analyse the orientation of grating-like laser-ablated ripples on the surface of Si (Figure 6) [20]; such ripples with a changing orientation can also be inscribed inside transparent materials [21]. Ripples have periods comparable to or smaller than the focal diameter. Ripples were made via laser ablation with $E_{p}=10\;\mu$J energy pulses. They were focused using a cylindrical lens of f=80 mm at a position of 180 mm before an objective lens of numerical aperture NA=0.26 (Mitutoyo). A vertical line with a 1 mm width at $1/e^{2}$ (long axis) and a ∼5 μm wide (short axis) focal region were formed. The ablation width was ∼400 μm (Figure 6); the ablation threshold fluence of Si is ∼0.2 J/${cm}^{2}$. Ablation was carried out at a 100 kHz repetition rate with 10 pulses per micron along the scan at a speed of v=10 mm/s. Different rotation speeds of the λ/2-waveplate were used along the scan, resulting in different lengths of patterns for a full rotation of $180^{\circ }$ (Figure 6a). The period of ripples on Si was Λ≈0.8×λ [22], as expected for conductive, non-transparent samples (Figure 6b); it has the highest intensity in the FFT map. Such azimuthally patterned gratings are used to discriminate left-hand from right-hand circular-polarised light upon reflection. Here, we use them to study polarisation changes upon reflection of linearly polarised incident 405 nm laser light.

**Figure 7:**
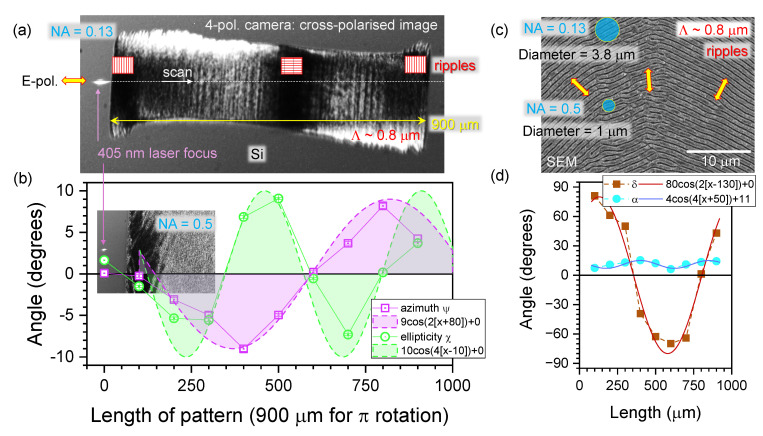
(**a**) Four-polarisation camera image of the azimuthally rotating pattern of ripples ablated under conditions of θ=π per 900 μm scan length of elliptical focus ∼400×5μ$m^{2}$. Scanning along the pattern was carried out in 100 μm steps and the polarimeter measured the Stokes vector at a 405 nm wavelength. (**b**) The azimuth ψ and ellipticity χ angles along the scan at NA=0.5 focusing conditions. The guidelines of qualitative fit are shown by ∼cos(2θ) and ∼cos(4θ) dependences. Inset shows the 4-pol. camera image with a laser focus on the non-ablated part of Si. (**c**) SEM images of azimuthally rotating ripples on Si show indicative sizes of focal spots at different NA focuses estimated as 1.22λ/NA. Fast-rotating polarisation was used for writing ripples. (**d**) The phase delay δ and auxiliary α angles along the ripple pattern.

The Stokes vector was determined from the reflection of 405 nm laser light linearly scanned along the central cross-section of the rotating ripple pattern (Figure 7). At this wavelength, Si has a high refractive index ($\tilde{n}=5.438+i0.342$) since it is close to the absorbance peak at 376 nm for direct transitions in Si, where $\tilde{n}\approx 6.7+i1.3$. The angles of azimuth and ellipticity were found qualitatively using the different angular dependence of the orientation angles of ripples, θ. The azimuth was close to 2θ dependence (expected for dichroism), while ellipticity was close to 4θ dependence (expected for birefringence). Also, the original orientation phases $\theta_{0}$ were perpendicular to each other for $\psi (\theta +\theta_{0})$ and $\chi (\theta +\theta_{0})$. Interestingly, only high-NA conditions (NA=0.5) were sufficient for collecting polarimetry data. This might be related to the larger collection angle important for the acquisition of anisotropic scattering. The high refractive index of Si (n≈3.5) facilitated strong reflection but equally a strong scattering. In the region of the ripples pattern, which is dark in the cross-polarised view, $\psi \approx \chi \to 0^{\circ }$, as was observed with the linear structures of the CFRP and polymer grating. Similar to the cases analysed above for linear patterns (Section 3.1), the phase δ was the largest and approached $80^{\circ }$ when α→0 (d). Such conditions correspond to linearly polarised reflected light.

Form birefringence of the pattern contributes to the phase changes of transmitted/reflected light. For the period Λ of ripples on the surface of the sample, the form birefringence is $\Delta n=n_{e}-n_{o}$ ($n_{e}$ is the refractive index of the extraordinary beam (or the fast axis) polarised perpendicular to the grooves and $n_{o}$ is for the ordinary beam (slow axis) along the grooves $n_{o}>n_{e}$). Form birefringence is negative by definition and contributes to the reflected phase [22] and corresponds to a uniaxial crystal with permittivity $\varepsilon_{\|}<\varepsilon_{⊥}$ with respect to the optical axis. For the depth of the structure *d*, the phase retardance is $\delta =\frac{2\pi }{\lambda }\Delta n\times 2d$ (a double path length due to reflection).

Apart from the form birefringence and anisotropic scattering, strong refractive index changes take place in the vicinity of the absorption bands with negative and positive dispersion regions; hence, ±Δn can occur at the neighboring wavelengths of the absorption spectral region. This affects the phase of the reflected light assessed via Stokes polarimetry. Since we used a 405 nm wavelength, absorption is usually present in transparent materials such as glasses and polymers. For the Lorentzian absorption spectral lineshape, the absorption $A\left(\omega \right)=\frac{\tau }{1+{\left(\omega -\omega_{0}\right)}^{2}\tau^{2}}$ and dispersion $D\left(\omega \right)=1-\frac{\left(\omega -\omega_{0}\right)\tau^{2}}{1+{\left(\omega -\omega_{0}\right)}^{2}\tau^{2}}$ are related to *k* and *n*, respectively; here, τ is the relaxation time (linked to the bandwidth of the line) and $\omega_{0}\equiv 2\pi c/\lambda_{0}$ is the cyclic frequency of the spectral position of the absorption line. Strong phase δ changes in polarimetric measurements due to dispersion n(λ) changes will appear as birefringence near the absorption band $\Delta n(\lambda_{0})$ with a sign dependent on the normal or anomalous side of the absorption line [23].

### 3.3. Diamond: A Transparent Sample with a Grating Pattern

The previous section analysed ripples on Si at a wavelength which is strongly absorbed and for the focal spot, which is comparable to or larger than the periodicity of the grating-like pattern. Next, we analyse nano-gratings defined by electron beam lithography (EBL) in a 5 μm thick poly-crystalline diamond membrane [24]. The structure is an optical spin–orbit converter—the q-plate—where the optical slow-axis rotates with azimuth angle θ according to β=q×θ. Such an optical element will generate an optical vortex beam with topological charge ϕ=±2q, i.e., the number of rotations along the propagation of a single wavelength. The most efficient spin–orbit conversion upon illumination with circularly polarised light occurs at a longitudinal phase retardance of π between E-field components along the grating pattern ‖ (extraordinary *e*-beam) and perpendicular ⊥ to it (ordinary *o*-beam). For such conditions, $\delta =2\pi (n_{e}-n_{0})h/\lambda$ for the height *h* of the structure. The form birefringence Δn defines the phase retardance and is dependent on the refractive index of the host material (here, diamond with n=2.4 at visible wavelengths) and the fill factor of the grating pattern.

**Figure 8:**
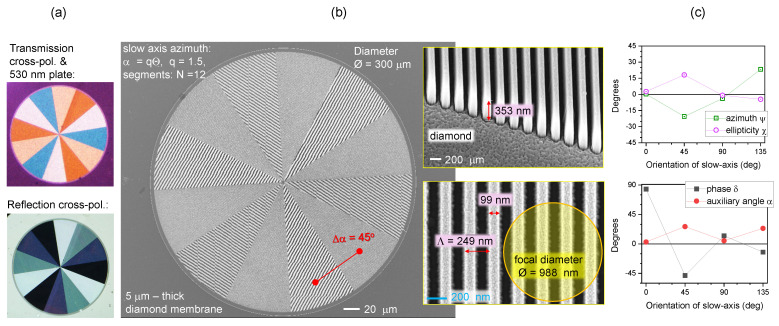
(**a**) Optical cross-polarised images of a q=1.5 q-plate in transmission and reflection modes. The optical slow-axis follows azimuth angle θ as β=qθ with N=12 segments (π/4 difference in the grating orientation between the neighboring regions). (**b**) SEM images of the q-plate showing period Λ=250 nm and with a 100 nm width of the diamond grating ridges; the poly-crystalline diamond membrane of 3μm thickness was Cr-coated, mask-patterned via EBL, developed and plasma etched (see processing details in ref. [25]). (**c**) The δ,α and ψ,χ dependencies from four segments in sequence measured in reflection using a polarimeter. Polarimetry was carried out using NA=0.5 focusing at a λ=405 nm wavelength.

Figure 8 shows data for the q=1.5 q-plate. The optical reflectance image shows distinct patterns in the cross-polarised image. The actual structure is shown in SEM images. The focal spot was approximately three times larger than the period Λ of the grating. The ellipticity and azimuth, as well as phase and auxiliary angles (c), followed the same trend as for the case of reflective larger-scale patterns on the CFRP and the Au-coated polymerised grating. In the case of the micro-membrane of patterned diamond q-plates, the sample was transparent. A large range of δ(θ) is suitable for detecting changes via polarimetry.

## 4. Conclusions and Outlook

Polariscopy analysis of a back-reflected laser beam (at a normal angle of incidence) is shown to be sensitive to surface anisotropy. From the Stokes vector, it is possible to calculate the delay phase δ, which is linked to the real part of the refractive index anisotropy and the auxiliary angle α, which defines the ratio of E-fields in s- and p-pol. The latter is related to absorption dichroism in the principle (s,p)- or (x,y)-plane. The back-scattered laser light had orientational dependence following 4θ angular dependence. Polarimetry provides a complimentary insight into surface anisotropy as compared to ellipsometry.

Such analysis of back-scattered/reflected signals using polarimetry is advantageous in industrial and engineering applications due to its simplicity compared to ellipsometry, which needs a lab-based setup and relies on a model of sample composition and structure (e.g., layer thicknesses). For polarimetry, rotation of polarisation is realised simply as a rotation of the sample. Linear polarisation is rotated by a λ/2-plate (or the linearly polarised laser itself in some cases) to access the orientation θ-dependence of the Stokes parameters. Remote sensing applications will benefit from the presented analysis of complex surfaces, such as CFRP, tested in this study. The four-polarisation camera can be used for anisotropy imaging and the determination of Stokes vectors, while the polarimeter provides a single-point measure from the reflected surface.

## Data Availability

The original contributions presented in the study are included in the article, further inquiries can be directed to the corresponding authors.
